# Self-assembling peptide haemostatic gel reduces incidence of pelvic collection after total mesorectal excision: Prospective cohort study

**DOI:** 10.1016/j.amsu.2021.102553

**Published:** 2021-07-09

**Authors:** Samuel Stefan, Mira Wagh, Najaf Siddiqi, Syed Naqvi, Emma Rawlinson, Anthony Shepherd, Jim Khan

**Affiliations:** a Colorectal Surgery, Queen Alexandra Hospital, Portsmouth Hospitals University NHS Trust, Portsmouth, UK; b University Hospitals Dorset, Poole, UK; c School of Sport, Health and Exercise Science, Faculty of Science and Health, University of Portsmouth, UK; d Professor of Surgery, University of Portsmouth, UK

**Keywords:** Pelvic surgery, Robotic, TME, Self assembling peptide

## Abstract

**Background:**

Pelvic surgery has the potential to leave behind a large raw surface, which can bleed and ooze postoperatively. The adoption of precision surgical approach for rectal cancers has led to reduction in blood loss. We aimed to assess 1) the feasibility and 2) the safety of using a self-assembling peptide (SAP) haemostatic agent (PuraStat®) after rectal cancer surgery to reduce the incidence of pelvic collections.

**Materials and methods:**

This prospective cohort pilot study compared the results of 25 consecutive cases of total mesorectal excision (TME) with use of 5–10 ml of SAP, and 25 consecutive cases without PuraStat® application (CON, control group). The groups were compared for complications (Clavien-Dindo grade III and IV classification), postoperative drain output and length of hospital stay (LOS). Statistical analysis was carried out using paired samples T test and Fisher's exact test

**Results:**

Fifty patients (SAP = 25, CON = 25) were enrolled into this study. Mean drain outputs (ml) on day 1, day 2 and day 3 were 60 ± 18, 89 ± 42 and 64 ± 45 in SAP group, and 102 ± 31, 95 ± 52, 66 ± 37 in CON group. This was significantly better for SAP group in day one after surgery. The mean LOS was shorter in SAP group (5.7 versus 7.4 days in CON, p 0.04). Clavien-Dindo III & IV complications were seen in two and five cases respectively (p 0.18). R0 resection rate (p 0.32) and lymph node harvest (p 0.13) were similar in both groups. There were no complications seen in relation to the application of the SAP.

**Conclusions:**

These initial data suggest that SAP is a safe product, and feasible to apply in the pelvis after TME surgery. It appears to shorten the LOS and reduce the postoperative drain output and may reduce the incidence of Clavien-Dindo grade III & IV complications.

## Highlights

• This is a first study to evaluate role of haemostatic agents in robotic colorectal surgery to reduce postoperative bleeding.
• We have demonstrated the use of this novel gel is associated with significantly reduced pelvic drain output after surgery.
• It has also highlighted a reduction in the length of stay, and possibly in the rate of postoperative complications.
• Further studies are necessary to establish the efficacy of prophylactic application of this gel after TME surgery for cancer.

## 1 Introduction

Pelvic surgery has the potential to leave behind a large raw surface, which can cause small amounts of capillary bleeding and oozing after surgery. These fluids are either absorbed by the peritoneum or can develop into a postoperative collection. Significant output from postoperative pelvic drains requires the drains to stay for a period of 24–72 h [[1], [2], [3]]. Keeping a surgical drain longer than 48 h could lead to a delay in discharging the patient from hospital and poor compliance with the enhanced recovery program (ERP) in colorectal surgery [4]. Furthermore, any infection in the area may convert this collection into an abscess, which may compromise anastomotic healing [5]. The adoption of precision surgical approach for rectal cancers has led to minimal blood loss intraoperatively; however pelvic drains are still needed after TME to avoid pelvic collection.

There are conflicting results in various studies [6,7] on whether to drain the pelvis or not. The situation is complex due to the heterogeneity of these studies and lack of adequate information with respect to level of lesion, level of anastomosis (extraperitoneal or intraperitoneal), type of drain used, use of preoperative therapy, indication for surgery (emergency or elective), intraoperative bleeding, and other risk factors [[1], [2], [3]].

Minor capillary oozing and lymphatic leakage may go unnoticed during surgery especially concealed by the pressure effect of the pneumoperitoneum, leading to the development of a pelvic hematoma or collection afterwards [2]. Therefore, there is a further need to use a preventative treatment and to assess its efficiency and safety.

Most conventional topical haemostatic agents obscure visibility once applied, making further surgical assessment of the area difficult. PuraStat® (3-D Matrix Europe SAS, France) is an inert, synthetic, transparent haemostatic hydrogel comprised of self-assembling peptides (SAPs), which spreads easily over uneven surfaces and hard to reach areas [8]. Previous studies in resectional endoscopy [[8], [9], [10]], ENT - ear, nose and throat surgery [11] and cardiothoracic surgery [[12], [13], [14], [15]] have revealed a significant reduction in the post-procedural complications with use of this product which reduces the oozing from capillaries and prevents secondary haemorrhage. The potential usage of a transparent SAP in colorectal resections has not been studied and presented so far.

We hypothesised that a prophylactic application of a SAP on the pelvic raw area may prevent fluid oozing and bleeding. This study intends to explore the safety and feasibility of application of SAP, with the aim to reduce the postoperative complications, LOS and drainage following surgery for rectal cancer.

## 2 Materials and methods

Patients with rectal cancer requiring TME surgery were enrolled into a prospective pilot cohort study. The cohort was divided into two groups comparing the group SAP with 25 consecutive patients receiving application of PuraStat® at the end of the procedure to the pelvic raw area, and a control (CON) group of 25 patients without Purastat® application. The patients were recruited over a period of four months (06–09/2019) and included in the study if they had a diagnosis of rectal cancer, were non-vulnerable adults, and willing and able to provide fully informed written consent for participation in the study and for the application of the SAP. Patients requiring multi-visceral resections, palliative resection, or having metastatic disease at presentation were excluded from the study. The project was registered with researchregistry.com with the number researchregistry6505 [16].

All patients underwent robotic rectal resection with TME surgery, with a standardised technique following the colorectal multidisciplinary team decision (MDT). They were managed post-operatively in an ERP setting. All patients had mechanical bowel preparation preoperatively and antibiotics at induction. A 20 Fr. drain was placed routinely in the pelvis. Drains were removed during the third postoperative day.

These two groups were compared for postoperative drain output and recovery parameters, complications (morbidity and mortality), and LOS. The postoperative complications were graded using the Clavien-Dindo classification [17]. Prolonged ileus was defined as lack of bowel or stoma function for more than three days after surgery. Anastomotic leak was defined as radiological or clinically palpable defect in the anastomosis. A pelvic collection or abscess was diagnosed on a computed tomography (CT) scan. CT scans were carried out for the patients who had prolonged ileus (>72 h), raised inflammatory markers (CRP>250) or clinical features of sepsis.

### 2.1 SAP application

**Fig. 1:**
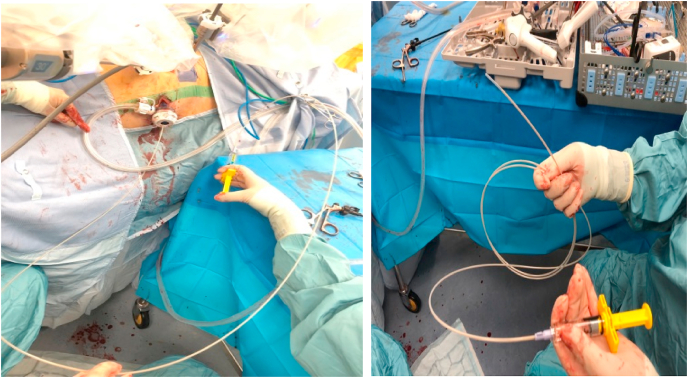
Preparation (right side photograph) and injection (left) of the haemostatic gel.

The nursing team were trained on the preparation of the product. An endoscopic applicator (E-Type nozzle system, Top Corporation, Japan) was used to allow the surgeon at the console to manage the application of PuraStat® with the robotic instruments in a controlled fashion (Fig. 1). A paintbrush technique was used to apply a thin layer of SAP over the pelvic raw area at the end of the procedure; the volume required was between 5 and 10 mL, and the injection was made by the bedside assistant but controlled by the operating surgeon using the robotic instruments.

**Fig. 2:**
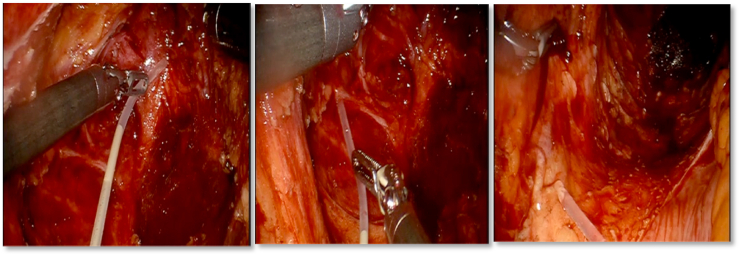
Application of PuraStat® in the pelvis - intraperitoneal view.

The product is ready-to-use in a prefilled syringe and it is deployed as a physical barrier to mitigate oozing. It is applied ergonomically in the pelvis and spread in a thin layer to cover the pelvic raw surface (Fig. 2).

**Fig. 3:**
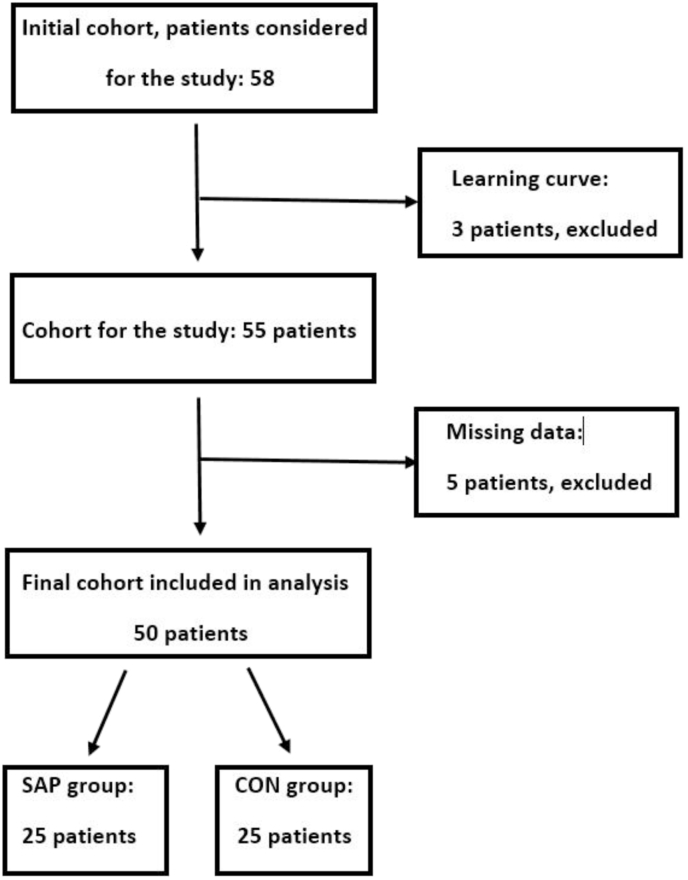
Consort flow chart of the study.

Three learning curve cases were undertaken to develop this application technique, to understand the volume of SAP required and thus to standardise the application method. These three patients were consented for the study but not included in the analysis of this pilot study, as per consort flow chart (Fig. 3).

This cohort study presents original research and is compliant with the STROCSS criteria checklist (Strengthening the reporting of cohort studies in surgery) [18].

### 2.2 Data analysis

Statistical analysis used IBM SPSS® Statistics for Windows, Version 24 (IBM, Chicago, IL). The distribution of data was assessed using descriptive methods (skewness, outliers, and distribution plots) and inferential statistics (Shapiro-Wilk test).Where there was no normal distribution, non-parametric analyses were performed. Data are presented as mean (SD). Paired samples T test was used for numerical data, with p value of <0.05 as significant, and Fisher's exact test was used to compare categorical data if the total sample size was small.

## 3 Results

A total of 50 patients completed the study. Patient demographics were similar between the two groups with regards to age, gender and BMI (Table 1). Mean age was 65 ± 11 years and 67 ± 13 years in SAP and CON groups respectively, with a mean BMI of 26.6 ± 5 and 26 ± 4.

**Table 1:** Patient characteristics.

	SAP (n = 25)	CON (n = 25)	P value
Mean age (range)^a^	65 ± 11 (32–88)	67 ± 13 (33–84)	0.48
Male/female	60/40	56/44	0.25
Mean BMI (range)	26.6 ± 5 (19–37)	26 ± 4 (19–37)	0.73
ASA I & II	16 (64%)	23 (92%)	0.49
ASA III & IV	9 (36%)	2 (8%)	
Tumour stage: T1 & T2 T3 & T4	10 (40%) 15 (60%)	9 (36%) 16 (64%)	0.42
N0 N1 & N2	11 (44%) 14 (56%)	7 (28%) 18 (72%)	0.10

a Data are presented as mean ± standard deviation, or as a percentage. N.B. BMI: Body Mass Index, ASA: American Society of Anesthesiologists.

Neo-adjuvant radiotherapy for downstaging was given to three patients in SAP, and to four in CON group. Both groups were comparable for tumour stage and nodal involvement.

**Fig. 4:**
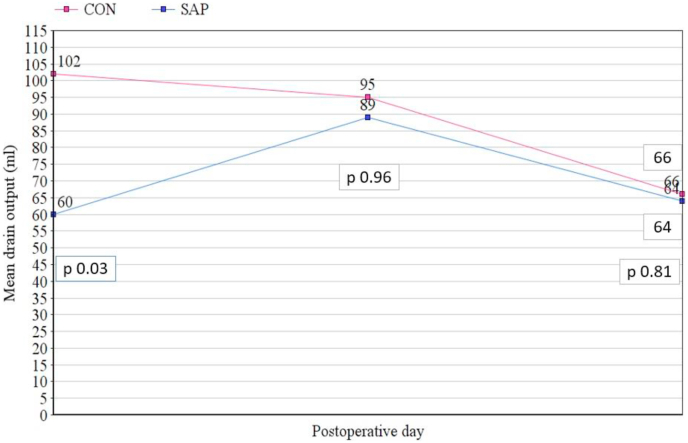
Comparison of the drain output during the first three days after surgery.

The recovery parameters included measurements of the drain output and the length of hospital stay. Mean drain outputs on day 1, day 2 and day 3 were 60 ± 18 ml, 89 ± 42 ml and 64 ± 45 ml in SAP group, and 102 ± 31 ml, 95 ± 52 ml, 66 ± 37 ml in CON group (Fig. 4), with a significant difference in day 1.

The surgical approach was a single docking standardised robotic TME surgery using daVinci® platform. IMA and IMV were divided and splenic flexure fully mobilised followed by TME in the pelvis down to the pelvic floor. Specimen was extracted via a Pfannenstiel incision and an end-to-end stapled colorectal anastomosis was fashioned [19].

The majority of cancers were locally advanced (T3 and T4) and required a low or ultra-low resection with a high-risk bowel anastomosis, hence a temporary stoma was considered (Table 2).

**Table 2:** Postoperative outcomes.

	Group 1 (n = 25)	Group 2 (n = 25)	P value
Mean operating time (min)^a^	261 ± 39	270 ± 79	0.57
Conversion to laparotomy	0	0	
Temporary stoma rate	13 (52%)	14 (56%)	0.78
Length of stay (days)	5.68	7.44	**0.04**
Clavien-Dindo grade III/IV	2 (8%)	5 (20%)	0.18
Compliance with ERP	20 (80%)	19 (76%)	0.23
R0 resection rate	24 (96%)	24 (96)	0.32
Lymph node harvest	33	26	0.13

a Data are presented as mean (SD), numbers, or as a percentage.

The mean time needed for the application of the SAP was 5 min (range 4–10 min). The mean operating time for SAP was 261 ± 39 min and 270 ± 79 min for CON (p 0.57). Both groups had a mean intraoperative blood loss of 20 ml (range 10–50 ml). There were no conversions to open surgery in both groups.

The mean LOS was 5.68 days for patients in SAP group and 7.44 days in CON group; this difference was statistically significant, p = 0.04 (Table 2). There was no mortality within 90 days after surgery.

The oncological outcomes were not different between SAP and CON for R0 resection (p 0.32) and lymph node count (p 0.13).

Postoperative grade III/IV Clavien-Dindo complications [17] were seen in two cases in SAP group and in five cases in CON group (Table 2, Table 3). The pelvic abscesses were diagnosed on postoperative CT scan performed if clinically indicated.

**Table 3:** Postoperative morbidity.

	SAP (n = 25)	CON (n = 25)	P value
Postoperative ileus	2	5	0.18
Pelvic abscess/collection	3	5	0.42
Anastomotic leak	1	1	n/a
Readmission	6	4	0.49
Reoperation	0	2	0.16

## 4 Discussion

To date, this is the first study to assess the use of a fluid haemostatic product in colorectal surgery. Specifically, this original research compared the use of a SAP with a control group undergoing rectal cancer operations. We hypothesised a no difference between the surgical outcomes in these groups and we found a series of statistically significant differences related to the surgical drain output and the LOS.

Precision oncological surgery is becoming the new paradigm in cancer management. In the era of minimally invasive surgery the perioperative blood loss is minimal. Newer tools allow surgeons to dissect better, without bleeding, and to avoid complications. The pelvic raw area after rectal cancer surgery can be a source of continuous oozing and lympho-vascular fluid can accumulate in the pelvis as postoperative hematoma/fluid collections. These could subsequently become infected, with formation of abscesses and leaks. For this very reason the routine use of pelvic drains after rectal cancer surgery has not been completely abolished [4].

The drain volume after rectal cancer surgery is dependent on various factors including surgical technique, factors related to the patients (obesity, narrow pelvis, and use of anticoagulants), and factors related to the cancer - such as tumour stage, size, and preoperative radiotherapy, and development of postoperative complications [4]. The mean drain volume in this study was significantly lower in the first postoperative day for the patients treated with SAP. Similarly, the length of hospital stay was shorter which probably reflects successful adherence to the ERP.

PuraStat® is a biocompatible, synthetic, self-assembling peptide haemostatic hydrogel; it is an inert, sterile solution, hence the risk of postoperative infection caused by the gel should be minimal. Our data did not suggest any increased risk of abscess formation or sepsis in the group treated with SAP application.

These SAPs form a three-dimensional nanofibre matrix when exposed to ionic fluids [8]. PuraStat is approved and CE marked for haemostasis during surgery in oozing bleeding in parenchyma of solid organs, vascular anastomoses and small vessels and capillaries in the gastrointestinal tract; it is additionally indicated for reduction of delayed bleeding following colonic ESD - endoscopic submucosal dissection [[8], [9], [10]]. We have considered the use of SAP only for the haemostatic purpose, and not for other reasons. As mentioned in the methodology, the aim of this study was to assess the ability of the gel to reduce the postoperative bleeding/oozing leading to the development of pelvic collection.

In 2006 Ellis-Behnke RG et al. published for the first time an animal experiment and demonstrated the use of a synthetic, non-toxic and non-immunogenic self-assembling peptide that forms a nanofibre barrier to establish immediate haemostasis when applied to open wounds [20]. PuraStat® has also been used in nasal endoscopic procedures, endoscopic resections in the GI tract and cardiac surgeries with promising results [[8], [9], [10], [11]]. Apart from preventing primary oozing and leakage, the use of haemostatic agents has been described to prevent a secondary haemorrhage. Most of the studies in this context originate from vascular surgery where the risk of postoperative bleeding is high [[12], [13], [14], [15]].

Masuhara et al. further established the efficacy of this agent in achieving intra-operative haemostasis in humans when applied to vessel-to-vessel anastomotic site. They reported no adverse effect and no post-operative bleed and suggested that this agent might be safer over other human or animal derived topical haemostatic agents who carry an inherent risk of transmitting infection and causing hypersensitivity reactions [14]. In another prospective non-randomised study of PuraStat® in cardiac surgery, Giritharan et al. showed similar results in terms of safety and efficacy of this agent [12]. They reported that the post-operative use of blood products in their series was below that of national average. Analysis of questionnaire given to ten operating surgeons scored this agent high in various subcategories including ease of application, time to coagulation, cross compatibility with other haemostatic agents and transparent nature of PuraStat® which does not obscure vision, thereby allowing further assessment of the operative field [13]. The drawbacks of this study are the lack of randomisation, and absence of patient stratification. The ongoing SASH trial (Strategy for aortic surgery haemostasis), which is in it a recruitment phase, is expected to provide a better level of evidence with regards to the haemostatic potential of this agent, in view of its randomised nature and focused patient selection [15].

In 2017, Lee et al. reported the efficacy of PuraStat® in achieving haemostasis in 60 patients undergoing endo-nasal powered turbinoplasty [11]. Subramaniam et al. in their study of 100 patients undergoing ESD and endoscopic mucosal resections at various sites reported a good haemostatic efficacy, even in patients on anticoagulation with an acceptable delayed bleeding rate of 3%. They also pointed out towards rapid onset of action of PuraStat® and reduction in use of electrocautery to achieve haemostasis [9,10]. The most recently publication from this group compares the use of this SAP to cautery for oozing bleeding, in a pragmatically designed RCT of high risk oesophageal and colonic ESD, with 101 patients undergoing ESD in a single centre. They report a haemostatic efficacy of 92.6%, with a delayed bleed rate of 4.3% [9].

De Nucci et al. (2020) have recently published data of their 3-centre observational study, using PuraStat® in acute gastrointestinal bleeds, on a cohort of 77 patients, over 2 years. They report a 90.3% haemostatic efficacy, with failure in 7/13 spurting bleeds. The re-bleed rate reported is 10.3% which compares well with the literature in this cohort [21]. Kondo et al. published their results of the use of a similar first generation self-assembling synthetic peptide named PuraMatrix® in achieving haemostasis following surgery for rectal cancer. They reported significant reduction in postoperative drainage in 10 patients, compared to 10 controls [22]. Our pilot study for application of SAP in colorectal cancer surgery revealed a significant reduction in drain output for the first postoperative day (Fig. 4).

The study also assessed the feasibility of application of SAP in the pelvis after robotic TME surgery. We evaluated the ease of application and standardised the technique while assessing any effects or complications from its use. Although there were more patients with readmission in SAP group, they only needed management for Clavien-Dindo grade I/II complications (Table 2, Table 3). The pelvic collections in SAP group were managed conservatively, whilst two patients in CON group had interventional radiology drainage. Most of the postoperative complications in the CON group occurred during the index admission. Two re-admissions led to re-operation due to postoperative abscess: abdominal washout and defunctioning ileostomy in one case, and a Hartmann's procedure for anastomotic leak in the second case were carried out (Table 3).

The limitations of this original research study include a small sample size and lack of randomisation. RCT design to assess further the effects of this intervention is required. It might be difficult to blind the operating team with a placebo product; however blind assessors of the outcome measures could be used. The outcomes of a potential RCT shall point towards routine use of a SAP as standard of care, and further a tailored application of SAP to any particular surgical case.

Another limitation of this pilot study is represented by inclusion of patients who had preoperative radiotherapy to the pelvis for downstaging purpose. Although a limited number of patients had neoadjuvant radiotherapy (3, respectively 4), this is a known risk factor for increased drainage output due to the presence of irradiated scarred tissue [4].

A recent review has also mentioned other potential uses of SAP in surgery, namely surgical haemostasis, haemostasis for radiation-induced proctitis, promotion of wound healing and tissue regeneration, and reduction of postoperative adhesions [23]. Future extended studies should take into consideration some of these new parameters as potential beneficial outcomes for the patients undergoing TME for cancer.

## 5 Conclusions

Use of SAP is safe and is associated with significantly reduced drain output after rectal cancer surgery and reduction in length of stay, and possible reduction in post operative complications. Further studies are necessary to establish the efficacy of prophylactic application of SAP after TME surgery.

## Ethical Approval

Granted - The study involves use of an inert, non-biological, product that has already been used in practice in other medical specialties: Gastroenterology, Endoscopy, ENT, Cardio-vascular Surgery, and with no reports of adverse reactions.The study has been registered with the local Audit Department with the registration number 4949, and has been brought to the local Research & Development Department.

## Funding

This work was supported by 3-D Matrix Europe SAS that provided the PuraStat® product and the applicator needed for this pilot study. The company representative has also provided support by reviewing the manuscript on one occasion.

## Author contribution

Please specify the contribution of each author to the paper, e.g. study design, data collections, data analysis, writing. Others, who have contributed in other ways should be listed as contributors. Jim Khan – conceptualization, methodology, writing – review & editing, supervision, Samuel Stefan – data curation, formal analysis, writing original draft, Najaf Siddiqi – supervision, Syed Naqvi – manuscript review, Emma Rawlinson – data curation, Mira Wagh – writing - literature review, references, Anthony Shepherd – writing – review & editing, validation.

## Guarantor

The Guarantor is the one or more people who accept full responsibility for the work and/or the conduct of the study, had access to the data, and controlled the decision to publish. Please note that providing a guarantor is compulsory.

## Declaration of competing interest

None.

## References

[1] Zhang H.Y., Zhao C.L., Xie J., Ye Y.W., Sun J.F., Ding Z.H. (2016 May). To drain or not to drain in colorectal anastomosis: a meta-analysis.

[2] Denost Q., Rouanet P., Faucheron J.L., Panis Y., Meunier B., Cotte E. (2017). To drain or not to drain infraperitoneal anastomosis after rectal excision for cancer: the GRECCAR 5 randomized trial. French research group of rectal cancer surgery (GRECCAR). Ann. Surg..

[3] Rondelli F., Bugiantella W., Vedovati M.C., Balzarotti R., Avenia N., Mariani E. (2014 Feb). To drain or not to drain extraperitoneal colorectal anastomosis? A systematic review and meta-analysis. Colorectal Dis..

[4] King P.M., Blazeby J.M., Ewings P., Franks P.J., Longman R.J., Kendrick A.H. (2006 Mar). Randomized clinical trial comparing laparoscopic and open surgery for colorectal cancer within an enhanced recovery programme. Br. J. Surg..

[5] Chambers W.M., Mortensen N.J.M. (2004 Oct). Postoperative leakage and abscess formation after colorectal surgery. Best Pract. Res. Clin. Gastroenterol..

[6] Guerra F., Giuliani G., Coletta D., Boni M., Rondelli F., Bianchi P.P. (2018). A meta-analysis of randomised controlled trials on the use of suction drains following rectal surgery. Dig. Surg..

[7] Cavaliere D., Popivanov G., Cassini D., Cirocchi R., Henry B.M., Vettoretto N. (2019 Jun). Is a drain necessary after anterior resection of the rectum? A systematic review and meta-analysis. Int. J. Colorectal Dis..

[8] Pioche M., Camus M., Rivory J., Leblanc S., Lienhart I., Barret M. (2016). A self-assembling matrix-forming gel can be easily and safely applied to prevent delayed bleeding after endoscopic resections. Endosc. Int. Open.

[9] Subramaniam S., Kandiah K., Chedgy F., Fogg C., Thayalasekaran S., Alkandari A. (2021 Jan). A novel self-assembling peptide for hemostasis during endoscopic submucosal dissection: a randomized controlled trial. Endoscopy.

[10] Subramaniam S., Kandiah K., Thayalasekaran S., Longcroft-Wheaton G., Bhandari P. (2019). Haemostasis and prevention of bleeding related to ER: the role of a novel self-assembling peptide. United European Gastroenterol J.

[11] Lee M.F., Ma Z., Ananda A. (2017). A novel haemostatic agent based on self-assembling peptides in the setting of nasal endoscopic surgery, a case series. Int J Surg Case Rep.

[12] Giritharan S., Salhiyyah K., Tsang G.M., Ohri S.K. (2018). Feasibility of a novel, synthetic, self-assembling peptide for suture-line haemostasis in cardiac surgery. J. Cardiothorac. Surg..

[13] Morshuis M., Schönbrodt M., Gummert J. (2019 April). Safety and performance of a self-assembling peptide haemostat for the management of bleeding after left ventricular assist device implantation: outcomes of a post market clinical follow-up study. J. Heart Lung Transplant..

[14] Masuhara H., Fujii T., Watanabe Y., Koyama N., Tokuhiro K. (2012). Novel infectious agent-free hemostatic material (TDM-621) in cardiovascular surgery. Ann. Thorac. Cardiovasc. Surg..

[15] Dias R.R. (2019). Strategy for aortic surgery hemostasis (SASH trial). NCT03917862.

[16] https://www.researchregistry.com/browse-the-registry#home/registrationdetails/60144ba5b04958001cad40c5/.

[17] Clavien P.A., Barkun J., de Oliveira M.L., Vauthey J.N., Dindo D., Schulick R.D. (2009). The Clavien-Dindo classification of surgical complications: five-year experience. Ann. Surg..

[18] R.A. Agha, A. Abdall-Razak, E. Crossley, N. Dowlut, C. Iosifidis, G. Mathew, For the STROCSS Group. STROCSS 2019 Guideline: Strengthening the Reporting of Cohort Studies in Surgery. 10.1016/j.ijsu.2019.11.002 31704426

[19] Mykoniatis Ioannis, Siddiqi Najaf, Khan Jim (2020). Robotic low anterior resection. Diseases of the colon & rectum.

[20] Ellis-Behnke R.G., Liang Y.X., Tay D.K., Kau P.W., Schneider G.E., Zhang S. (2006 Dec). Nano hemostat solution: immediate hemostasis at the nanoscale. Nanomedicine.

[21] de Nucci G., Reati R., Arena I., Bezzio C., Devani M., della Corte C. (2020 Sep). Efficacy of a novel self-assembling peptide hemostatic gel as a rescue therapy for refractory acute gastrointestinal bleeding. Endoscopy.

[22] Kondo Y., Nagasaka T., Kobayashi S., Kobayashi N., Fujiwara T. (2014 Mar-Apr). Management of peritoneal effusion by sealing with a self-assembling nanofiber polypeptide following pelvic surgery. Hepato-Gastroenterology.

[23] Sankar S., O'Neill K., Bagot D'Arc M., Rebeca F., Buffier M., Aleksi E., Fan M., Matsuda N., Gil E.S., Spirio L. (2021 Jun). Clinical use of the self-assembling peptide RADA16: a review of current and future trends in biomedicine. Front. Bioeng. Biotechnol..

